# Fast-Disintegrating Oral Films Containing Nisin-Loaded Niosomes

**DOI:** 10.3390/molecules30183715

**Published:** 2025-09-12

**Authors:** Ali A. Amer, Yasir Karkar, Lewis Bingle, Amal Ali Elkordy, Cheng Shu Chaw

**Affiliations:** 1 School of Pharmacy and Pharmaceutical Sciences, Faculty of Health Sciences and Wellbeing, University of Sunderland, Sunderland SR1 3SD, UK; yasir.karkar@sunderland.ac.uk (Y.K.); amal.elkordy@sunderland.ac.uk (A.A.E.); hs0cch@sunderland.ac.uk (C.S.C.); 2 School of Nursing and Health Sciences, Faculty of Health Sciences and Wellbeing, University of Sunderland, Sunderland SR1 3SD, UK; lewis.bingle@sunderland.ac.uk

**Keywords:** nisin, niosomes, microfluidics, buccal peptide delivery, oral films, antimicrobial peptides, localized drug delivery, nanoparticle formulation, mucosal drug delivery

## Abstract

Nisin, a food preservative lantibiotic produced by *Lactococcus lactis*, exhibits potent antimicrobial activity against a wide range of Gram-positive pathogens, including antibiotic-resistant strains such as methicillin-resistant *Staphylococcus aureus* (MRSA). This study explores the development of a novel nano drug delivery platform comprising nisin-loaded niosomes, formulated via microfluidic mixing, and integrated into fast-dissolving oral films for targeted buccal administration. Microfluidic synthesis enabled the precise control of critical parameters including the flow rate ratio, surfactant composition, and lipid concentration, resulting in uniform niosomal vesicles with optimal size distribution (100–200 nm), low polydispersity index, and high encapsulation efficiency. Span 40 and Span 60 were employed as non-ionic surfactants, stabilized with cholesterol to improve bilayer rigidity and drug retention. The encapsulated nisin demonstrated improved physicochemical stability over time and protection against proteolytic degradation, thus preserving its antimicrobial potency. The niosomal suspensions were subsequently incorporated into polymer-based oral films as a final dosage form composed of polyvinyl alcohol (PVA) as the primary film-forming polymer, polyethylene glycol 400 (PEG400) as a plasticizer, and sucralose and mint as a sweetener and flavoring agent, respectively. A disintegrant was added to accelerate film dissolution in the oral cavity, facilitating the rapid release of niosomal nisin. The films were cast and evaluated for thickness uniformity, mechanical properties, disintegration time, surface morphology, and drug content uniformity. The dried films exhibited desirable flexibility, rapid disintegration (<30 s), and consistent distribution of nisin-loaded vesicles. In vitro antimicrobial assays confirmed that the bioactivity of nisin was retained post-formulation, showing effective inhibition zones (16 mm) against *Bacillus subtilis*. This delivery system offers a promising platform for localized antimicrobial therapy in the oral cavity, potentially aiding in the treatment of dental plaque, oral infections, and periodontal diseases. Overall, the integration of microfluidic-synthesized nisin niosomes into oral films presents a novel, non-invasive strategy for enhancing the stability and therapeutic efficacy of peptide-based drugs in mucosal environments. Physicochemical characterization of the niosomes and niosome films was performed using Fourier-transform infrared spectroscopy (FTIR), differential scanning calorimetry (DSC) and thermogravimetric analysis (TGA) to evaluate thermal stability and scanning electron microscopy (SEM) to assess surface morphology. In vitro peptide release studies demonstrated sustained release from both niosomal suspensions and film matrices, and the resulting data were further fitted to established kinetic models to elucidate the underlying drug release mechanisms. This delivery system offers a promising platform for localized antimicrobial therapy in the oral cavity, potentially aiding in the treatment of dental plaque, oral infections, and periodontal diseases. Overall, the integration of microfluidic-synthesized nisin niosomes into oral films presents a novel, non-invasive strategy for enhancing the stability and therapeutic efficacy of peptide-based drugs in mucosal environments.

## 1. Introductions

Niosomes are self-assembling, nanoscale vesicles that have gained significant attention in the field of drug delivery. Initially explored in cosmetic formulations in the late 1970s, these nanocarriers have since proven highly effective for pharmaceutical applications, especially for encapsulating both hydrophilic and hydrophobic drugs [1]. Niosomes are composed of non-ionic surfactants, typically combined with cholesterol, which form a bilayer structure. This bilayer allows niosomes to encapsulate hydrophilic drugs within their aqueous core and hydrophobic drugs within their lipid bilayer, making them versatile carriers for various therapeutic agents. The characteristics of niosomes, such as their size, stability, and drug release properties, can be controlled by adjusting factors like lipid composition, the method of preparation, and the type of drug encapsulated. These adaptable parameters make niosomes ideal for enhancing drug bioavailability, protecting sensitive compounds from degradation, and enabling controlled and sustained drug release.

**Figure 1:**
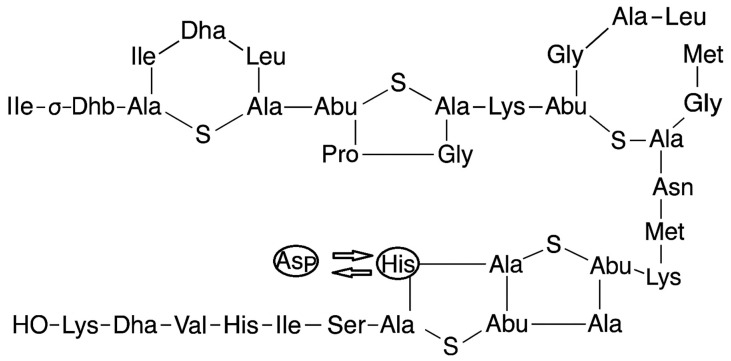
Nisin A and nisin Z structures. Histidine in nisin A is replaced by asparagine in nisin Z on the 27th amino acid.

Nisin A is a naturally occurring antimicrobial peptide that is classified as a bacteriocin and lantibiotic produced by *Lactococcus lactis* with a molecular weight of approximately 3.5 kDa and an isoelectric point (pI) of about 8.8, resulting in a net positive charge at physiological pH, enabling strong electrostatic binding to negatively charged bacterial membranes [2,3]. Its solubility is pH dependent around 57 mg/mL at pH 2, decreasing to ~1.5 mg/mL at pH 6, and only ~0.25 mg/mL at pH 8.5; similarly, its stability declines at higher pH due to chemical and structural modifications. Nisin maintains heat stability under acidic conditions but is vulnerable to enzymatic degradation and oxidation unless protected via encapsulation or embedding [4]. It is widely known for its potent activity against Gram-positive bacteria, including foodborne pathogens like *Listeria monocytogenes* and *Staphylococcus aureus* [5]. Nisin Z is the closest variant of nisin A, differing by a single amino acid substitution (histidine in nisin A is replaced by asparagine in nisin Z; Figure 1) [6]. This minor variation leads to a slight difference in solubility and diffusion characteristics, with nisin Z generally exhibiting better solubility at neutral pH [6]. Both forms share a common mechanism of action, targeting bacterial cell membranes by binding to lipid II, an essential component of cell wall synthesis, and forming pores that cause cell lysis and death [7]. Nisin’s structure is characterized by unusual amino acids, such as lanthionine and β-methyllanthionine, formed through post-translational modifications, which contribute to its stability in acidic environments and resistance to heat [8]. Nisin has been approved for use as a natural preservative in food by regulatory agencies like the FDA and EFSA due to its safety profile and effectiveness [9]. Beyond its application in food preservation, nisin has attracted attention in pharmaceutical research, particularly for its potential in treating antibiotic-resistant infections, such as MRSA and *Clostridium difficile* [10]. However, its therapeutic use is limited by rapid enzymatic degradation and a short half-life in biological systems. To overcome these limitations, advanced delivery methods like encapsulation in nanoparticles, niosomes, or liposomes are being explored to enhance its stability, bioavailability, and peptide release. This makes nisin a promising candidate for pharmaceutical applications, including oral, topical, and wound care formulations, especially in combating antibiotic-resistant bacterial infections [11]. The combination of nisin-loaded niosomes with fast-disintegration oral films introduces an innovative drug delivery platform. Fast-disintegration oral films (FDOFs) dissolve quickly in the mouth without the need for water, providing a convenient and rapid method for drug administration. This is particularly useful for patients with swallowing difficulties or those needing immediate therapeutic action. When nisin-loaded niosomes are incorporated into FDOFs, they offer an efficient way to deliver nisin locally in the oral cavity or systemically, ensuring rapid disintegration and quick onset of action. This formulation holds great promise for treating bacterial infections in the mouth or gastrointestinal tract, as well as for enhancing patient compliance and comfort. Through niosomal encapsulation and oral film technology, the therapeutic potential of nisin can be enhanced and offering a new and effective method for drug delivery.

## 2. Application of Nisin Loaded Niosomes

Nisin-loaded niosomes represent a promising platform for the targeted delivery of antimicrobial agents within the oral cavity and potentially for systemic health benefits. Encapsulation of nisin in niosomes offers enhanced stability, protection from enzymatic degradation, and controlled release, thereby improving its therapeutic efficacy. When incorporated into fast-disintegrating oral films, this formulation facilitates localized and rapid delivery of nisin to mucosal surfaces, making it particularly effective for the treatment of oral infections such as dental caries, periodontitis, gingivitis, and oral candidiasis [12]. The anti-biofilm properties of nisin are further augmented by niosomal encapsulation, which is especially beneficial in managing persistent biofilm-associated infections in orthodontic patients or on dental prostheses [13]. Moreover, the use of nisin-loaded films in post-dental procedures may aid in preventing secondary infections and promoting wound healing through localized antimicrobial action. As an adjunct to conventional periodontal therapy, these formulations can enhance the reduction of microbial load and inflammation in periodontal pockets in addition to reducing plaque and gingivitis [14,15]. Additionally, nisin-based oral films may offer a convenient alternative to traditional mouthwashes, providing sustained antimicrobial activity in scenarios where rinsing is impractical. Earlier studies have demonstrated the advantages of microfluidic systems for producing nanocarriers with narrow size distributions and reproducible properties [16]. Similarly, oral film formulations have been explored as promising drug delivery systems due to their rapid disintegration and ability to bypass hepatic first-pass metabolism in addition to improving patient compliance and facilitate mucosal absorption [17]. However, there is a notable gap in integrating these two approaches. While microfluidic prepared nanocarriers have mainly been applied to parenteral routes, their incorporation into oral films for peptide delivery remains largely unexplored. In particular, limited attention has been paid to how these nanocarriers behave during film processing and storage also the influence of drug release profiles and mucosal permeation. Despite the potential of nisin as an antimicrobial peptide, its incorporation into practical oral dosage forms remains limited. While previous studies have explored dietary administration, mouth rinses, and tablets, no research has yet formulated nisin-loaded niosomes into an orodispersible film. This study addresses that gap by focusing on the pharmaceutical development and characterization of the formulation, including niosome stability, film integrity, and drug release behavior.

## 3. Aim and Objectives

The primary aim of this study is to prepare and evaluate niosomes encapsulated with nisin A as a potential antimicrobial agent against Gram-positive bacterial infections. The study seeks to compare the effectiveness microfluidic technology in the development of nisin-loaded niosomes. Ultimately this may lead to the formulation of fast-disintegrating oral films that can facilitate localized delivery of nisin A in the oral cavity.

To achieve this aim, several specific objectives will be pursued. First, the study will focus on the preparation of niosomes encapsulated with nisin A using both thin film hydration and microfluidic technology. The physicochemical properties of the prepared niosomes, including size, zeta potential, and encapsulation efficiency, will be thoroughly evaluated to determine the optimal formulation. Following this, a comparative analysis of the two production methods will be conducted to assess their effectiveness in producing niosomes with desirable characteristics for drug delivery. This analysis will also include stability assessments and antimicrobial activity evaluations against Gram-positive bacterial strains *Bacillus subtilis* as a convenient non-pathogenic model for common oral infections.

Subsequently, the best-performing niosome formulation will be utilized to develop fast-disintegrating oral films that allow for the quick release and localized delivery of nisin A. The formulation process will involve exploring various combinations of multiple polymers and super-disintegrating agents to optimize the oral film’s disintegration time and release profile. In vitro studies will be conducted to evaluate the disintegration and release characteristics of these films, ensuring they effectively deliver nisin A at the site of infection.

Lastly, the antimicrobial properties of the formulated products will be assessed to evaluate for their potential as innovative treatments for oral infections and to determine their effectiveness in preventing biofilm formation and promoting oral hygiene. By achieving these objectives, the study aims to contribute valuable insights into the development of nisin A-loaded niosomes and fast-disintegrating oral films as effective, targeted solutions for combating bacterial infections, particularly considering the growing challenge of antibiotic resistance.

## 4. Materials and Methods

### 4.1. Material

Nisin A was obtained from Molekula Group (Darlington, UK). Non-ionic surfactants, including Span 60 (sorbitan monostearate, S60), Span 40 (sorbitan monopalmitate, S40), along with cholesterol, polyvinyl alcohol (PVA Mw 31 kDa), and polyvinylpyrrolidone (PVP; Mw 360 kDa), were purchased from Merck (Darmstadt, Germany). Kolliphor^®^ RH40 (polyoxyl 40 hydrogenated castor oil, RH40), Kolliphor^®^ ELP (polyoxyl 35 castor oil, ELP), and polyethylene glycol 400 (PEG400) were supplied by BASF (Stockport, UK). Additional pharmaceutical excipients included sodium starch glycolate (SSG) GLYCOLYS^®^ from Roquette UK Ltd. (London, UK), microcrystalline cellulose (MCC) Avicel© from IFF Pharma Solutions (Shadsworth, UK), hydroxypropyl cellulose SSL (HPC-SSL) from nisso excipients (Düsseldorf, Germany), hydroxypropyl methylcellulose (HPMC) purchased from Merck (Darmstadt, Germany), and croscarmellose sodium (CCS) VIVASOL^®^ from JRS PHARMA (New York, NY, US). The standard Gram-positive bacterial strain *Bacillus subtilis* NCTC 3610 and all culture media were obtained from the microbiological repository of the Microbiology Laboratory, University of Sunderland. All chemicals and reagents used were of pharmaceutical grade and high purity.

### 4.2. Methods

#### 4.2.1. Niosomes Formulation

Five different niosomal formulations were prepared using the microfluidic technique. Various molar ratios of cholesterol (Ch), Span 60 (SP60), Span 40 (SP40), Kolliphor RH40 (RH40), and Kolliphor ELP (ELP) were mixed to assess their influence on vesicle characteristics. The total mass of each formulation varied depending on the molecular weights of the individual components, ranging between 120 mg and 140 mg for a combined 150 µmol of components. To standardize the formulations for comparison, each was scaled to a total mass of 40 mg by applying an appropriate scaling factor based on its initial total mass ensuring that the molar ratios remained consistent (Table 1). The choice of molar ratios was dependent on different experiments that were performed on vancomycin hydrochloride as a hydrophilic peptide drug [18]. All formulations were prepared in duplicate (*n* = 2) and tested in triplicate (*n* = 3) to ensure reproducibility and accuracy of the results.

#### 4.2.2. Microfluidic Niosome Synthesis

**Figure 2:**
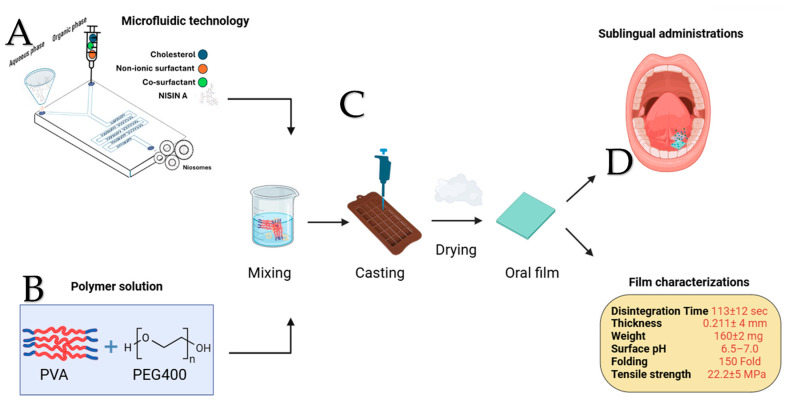
Development of nisin-loaded niosomal oral films via microfluidic synthesis (**A**), polymer solution preparation with PVA and PEG 400 (**B**), film casting and drying process (**C**), and final film evaluation for sublingual administration based on disintegration time, thickness, weight, surface pH, folding endurance, and tensile strength (**D**).

Niosomes containing nisin A were prepared using the microfluidic method (Precision NanoAssemblr™ Benchtop, Vancouver, BC, Canada) with disposable single-use microfluidic mixing cartridges (Number: 1142-050). Five different niosomal formulations were prepared, with the specific ingredient amounts listed in Table 1. The lipid and surfactant components were accurately weighed and dissolved in ethanol to create a homogeneous organic phase, while the aqueous phase consisted of 2 mg/mL nisin A in water. The cholesterol–surfactant solution was loaded into one syringe (organic phase), and the nisin A solution was loaded into another syringe (aqueous phase). Both syringes were inserted into the cartridge, which was surrounded by a heating block maintained at 60 °C to facilitate lipid hydration and vesicle formation. The solutions were injected through separate inlets into the microfluidic mixing chamber (as shown in Figure 2A), where they combined under controlled laminar flow. This precise mixing promoted the spontaneous formation of niosomes, encapsulating nisin A within the lipid–surfactant vesicles. The device was operated at a total flow rate (TFR) of 12 mL/min, maintaining aqueous-to-organic phase ratios of either 3:2 or 3:1, depending on the formulation. The resulting niosomes were immediately collected and stored at 4 °C to preserve stability. The formulations were subsequently characterized for particle size, polydispersity, and encapsulation efficiency, ensuring reproducibility across batches.

#### 4.2.3. Quantifications Methods

**Figure 3:**
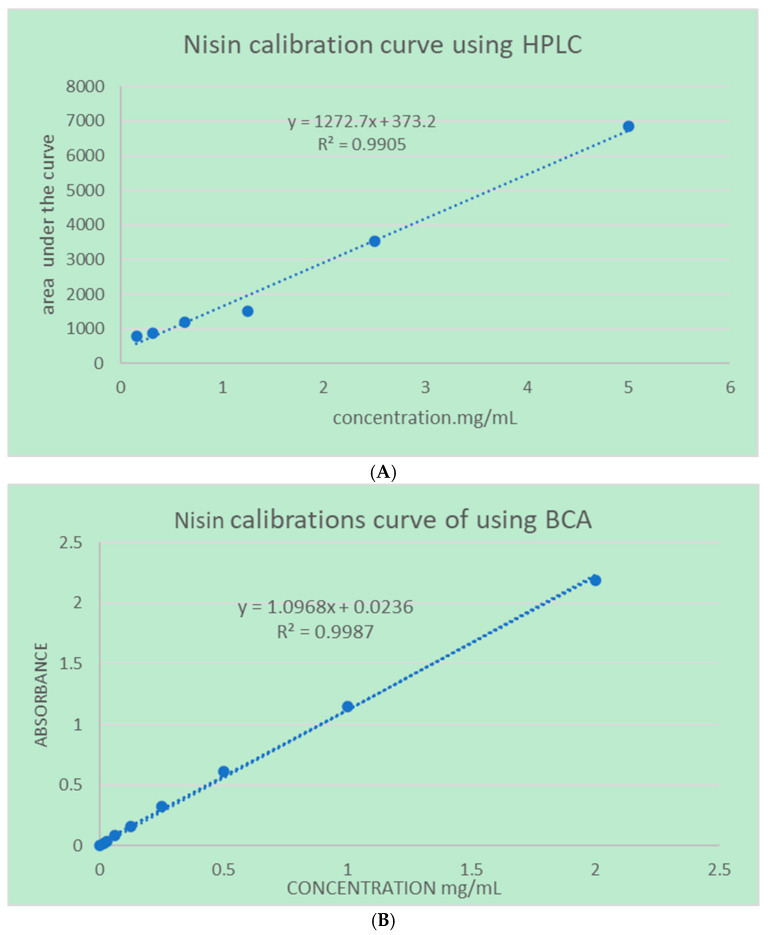
(**A**) The calibration curve constructed using HPLC analysis. (**B**) The calibration plot obtained using the BCA protein assay reagent.

To develop a calibration curve for nisin A, Agilent 1290 infinity HPLC system coupled with a G4212A diode array detector (DAD) was used (Agilent Technologies, Inc., Santa Rosa, CA, US), a series of standards were prepared at concentrations of 0.156, 0.3125, 0.625, 1.25, 2.5 and 5 mg/mL. The HPLC system was equipped with a super C18 analytical column (100 mm × 1.5 mm, 1.6 µm particle size) and operated with a mobile phase composed of water containing 0.05% trifluoroacetic acid (mobile phase A) and acetonitrile containing 0.05% trifluoroacetic acid (mobile phase B). A gradient elution was used, starting with 100% A and 0% B, linearly changed to 0% A and 100% B over 5 min, then holding 100% B for 1 min, before returning to the initial conditions and re-equilibrating. The flow rate was set at 0.3 mL/min, the column temperature was maintained at 28 °C, and the detection was performed at 210 nm. Calibration standards were prepared by dissolving nisin A in water to make a stock solution of 50,000 µg/mL, followed by serial dilutions to achieve the desired concentrations. A 20 µL aliquot of each standard was injected, and the peak areas were recorded on 1.8 min retention time. The calibration curve was constructed by plotting the peak areas against the corresponding concentrations, and the linearity of the curve was assessed by calculating the correlation coefficient (R^2^) as shown in Figure 3. Detecting nisin at low concentrations using HPLC with UV detection presents challenges due to its structural characteristics. Nisin is a peptide that lacks strong chromophores, such as aromatic amino acids like tryptophan or tyrosine, which are responsible for significant UV absorbance at common detection wavelengths (e.g., 210–280 nm). As a result, nisin exhibits minimal UV absorbance, and the lower limit of quantification (LLOQ) is 0.274 µg/mL, leading to a poor signal-to-noise ratio and reduced sensitivity in UV-based HPLC detection methods. Due to this limitation, quantification of small amounts of nisin for drug release and dissolution analyses was conducted using the bicinchoninic acid (BCA) protein assay method. The analysis was carried out using the Thermo Scientific™ Pierce™ BCA Protein Assay Kit (Waltham, MA, US), following the test tube method protocol provided by the manufacturer [19]. This assay is based on the reduction of Cu^2+^ to Cu^+^ by chemical groups present in the sample under alkaline conditions, followed by the formation of a purple-colored complex with bicinchoninic acid, which is detectable at 562 nm. Serial dilutions of the sample were prepared and individually mixed with the BCA working reagent, which was freshly prepared by combining Reagent A and Reagent B in a 50:1 ratio, as per the manufacturer’s instructions. The reaction mixtures were incubated at 37 °C for 30 min, after which the absorbance was measured at 562 nm using a UV–Vis spectrophotometer Drawell DU-8600R (Drawell Scientific Instrument, Chongqing, China) Quantification was based on direct comparison of absorbance values across the sample set 0.0156, 0.03125, 0.0625, 0.125, 0.25, 0.5, 1, 2 mg/mL All calibration points for both concentration–AUC and concentration–absorbance curves were prepared and analyzed in triplicate to ensure the reliability and reproducibility.

### 4.3. Niosome Characterization

The characterization of niosomes was performed to assess their size, morphology, and encapsulation efficiency (EE). Size distribution was determined using Zeta-sizer device (Malvern Instrument Ltd., Malvern, UK), where 50 times dilution of sample that contains niosomal suspension was analyzed at room temperature. The average hydrodynamic size (in nm) and polydispersity index (PDI) were recorded, providing insights into the size and uniformity of the niosomes. For morphological analysis scanning electron microscope (SEM), a HITACHI S-3000N scanning electron microscope (Tokyo, Japan) was used to observe surface characteristics by spreading a sample on a metal stub, drying it under vacuum, and coating it with iridium to reveal the shape and structure of the niosomes. Encapsulation efficiency (EE) was evaluated by first separating the unencapsulated nisin A using centrifugation for 1 h at 15,000 rpm and 4 °C. The clear supernatant solutions were collected, and the encapsulated drug was quantified using HPLC for the free drug in the supernatant. In addition, the amount of encapsulated drug was measured for few formulations to confirm that the amount of the drug in supernatant matched with the amount in pellet. The encapsulation efficiency was calculated by comparing the amount of unencapsulated drug to the total drug initially added, providing a measure of the formulation’s effectiveness in drug incorporation. The encapsulation efficiency was calculated according to Equation (1). These characterization techniques ensure that the niosomes meet the desired criteria for size, morphology, and drug loading, essential for their intended therapeutic application.

(1) EE%=((Total drug−Free drug)/Total drug)×100

### 4.4. Antibiotic Assay Using an Agar Diffusion Method

To assess the antibacterial effectiveness of nisin-loaded niosomes, an antibiotic assay was conducted using the agar diffusion method against the Gram-positive bacterium, *Bacillus subtilis*. Initially, a bacterial culture of *Bacillus subtilis* was cultivated in nutrient broth and incubated overnight at 37 °C to reach its exponential growth phase. Once ready, the culture was transferred to a tube containing sterile saline and thoroughly vortexed to ensure homogeneity. The bacterial suspension was then standardized to a turbidity equivalent to the 0.5 McFarland standard, using a bio-DEN-1 densitometer (Swindon, UK). Next, nutrient agar plates were prepared by pouring molten agar into sterile Petri dishes and allowing it to solidify. After the agar had set, the surface was uniformly inoculated with the *Bacillus subtilis* culture using a sterile cotton swab. Different formulations of nisin-loaded niosomes were then prepared and centrifuged to form a pellet. This pellet was washed twice to eliminate any unencapsulated nisin residues and was subsequently dispersed onto sterile paper disks in a designated volume. Control samples were also prepared, which included free nisin 2 mg/mL solution and blank niosomes (those without the drug). Sterile paper discs, each measuring 6 mm in diameter, were impregnated with 20 µL of the nisin-loaded niosome suspension. The nisin concentrations for niosomes formulation are dependent on entrapment efficiency, the free nisin solution, and the blank niosomes. These discs were placed on the inoculated agar plates and left at room temperature for 15–20 min to allow the drug to diffuse into the agar medium. Following this, the plates were incubated at 37 °C for 24 h, permitting bacterial growth and the formation of inhibition zones. After incubation, the zones of inhibition (clear areas surrounding the discs where bacterial growth was prevented) were measured with a ruler. The diameter of each inhibition zone was recorded in millimeters, providing a quantitative assessment of antibacterial activity. The efficacy of the nisin-loaded niosomes was evaluated by comparing these zones with those produced by free nisin and blank niosomes. Larger inhibition zones indicated greater antibacterial potency, highlighting the potential of niosomal formulations for improved drug delivery. This optimized agar diffusion method enables a thorough evaluation of the antibacterial effectiveness of nisin-loaded niosomes against *Bacillus subtilis*.

### 4.5. Film Optimization, Preparation, and Characterization Method

Fast-disintegrating oral films were prepared using the solvent casting technique to develop an effective and stable drug delivery system. Initially, a range of film-forming polymers such as polyvinyl alcohol (PVA), polyvinylpyrrolidone (PVP), hydroxypropyl methylcellulose (HPMC), and hydroxypropyl cellulose (HPC) were individually evaluated for their suitability in forming uniform, flexible films. Each polymer (5% *w*/*w*) was dissolved in distilled water under continuous magnetic stirring, with mild heating applied as necessary to facilitate complete dissolution. Polyethylene glycol 400 (PEG 400) was incorporated at a concentration of (1% *w*/*w*) as a plasticizer to enhance film flexibility and reduce brittleness. To improve organoleptic properties, sweetener and flavoring agent (1% *w*/*w*) were also added. The prepared niosomal suspension (3.5% *w*/*w*) was subsequently introduced into the polymeric solution under gentle stirring to ensure homogenous drug distribution. Upon visually evaluating the physical characteristics of the films including appearance, transparency, uniformity, flexibility, and peelability, the polymer that yielded the most desirable structural properties was selected for further formulation refinement. In the subsequent optimization phase, this selected polymer was combined with one of three disintegrating agents, including microcrystalline cellulose (MCC), sodium starch glycolate (SSG), or croscarmellose sodium (COS), each incorporated at 1% *w*/*w* to distilled water. The resulting film-forming solutions were cast into uniformly levelled molds, and the film was dried under fume hood at room temperature (25 °C) under ambient laboratory humidity for 24 h until the solvent evaporated. Films were prepared into standardized dimensions (3 × 2 cm) suitable for single-dose administration and stored in sealed containers under low humidity, protected from light as shown in Figure 2C. The uniformity of film weight across different batches was evaluated using an analytical balance, and the mean values along with standard deviations were calculated to confirm batch-to-batch consistency. Film thickness was determined at four separate points using a micrometer screw gauge (Philip Harris, Accrington, UK) to verify uniform structural integrity and drying. Film flexibility was assessed using a hand-folding test, wherein the number of folds required before the film exhibited cracking was recorded. The tensile strength of the films was measured using a digital force tester (Chatillon CS2+, Berwyn, PA, USA) attached to a texture analyzer, quantifying the maximum stress the film could withstand before breaking and the maximum strain at the break. The deformation rate was 20 mm/minute, and the data were collected every 50 milliseconds. To ensure suitability for oral application, the surface pH and the pH of the film-dissolution medium were assessed by immersing film samples in distilled water and measuring the pH with universal pH indicator strips (Simplex Health, Philadelphia, PA, USA), with a neutral pH considered optimal to avoid mucosal irritation. Lastly, the in vitro disintegration time was determined by immersing individual film strips in 10 mL of deionized water maintained at 37 °C, recording the time required for complete disintegration. 

### 4.6. Fourier Transform Infrared Spectroscopy (FTIR)

FTIR analysis was performed to investigate the compatibility between nisin and the excipients used in the film formulation. Spectra were recorded using an Agilent Cary 630 FTIR spectrometer (Agilent Technologies, Inc., Santa Rosa, CA, USA) equipped with a deuterated triglycine sulfate (DTGS) detector and operated in % transmittance mode. Samples were prepared by directly placing a small quantity of the samples onto the sample holder without further modification. Each sample was scanned over a wavenumber range of 700 to 4000 cm^−1^ using the SqrTriangle apodization function. A total of 32 scans were collected per sample to improve signal-to-noise ratio, with a spectral resolution of 2 cm^−1^. The resulting spectra were smoothed and baseline-corrected using IRsolution 1.6 software. Key functional group peaks were identified and compared between the spectra of pure nisin and the formulated film to assess any potential shifts, disappearance, or appearance of new peaks, which may indicate interactions between the drug and excipients. 

### 4.7. Thermal Analysis

#### 4.7.1. Thermogravimetric Analysis (TGA/DTG)

Thermogravimetric analysis (TGA) was performed to evaluate the thermal stability and moisture content of the film formulations. Approximately 5–10 mg of each sample (nisin-only film and nisin-loaded niosomal film) was placed in a platinum crucible and analyzed using a METTLER Thermal Analyzer (Mettler Instrumente AG, Greifensee, Zurich). The heating program was set from 25 °C to 600 °C at a constant rate of 10 °C/min under a nitrogen atmosphere (flow rate: 20–40 mL/min) to prevent oxidative degradation. The resulting TGA curves were used to determine the initial weight loss associated with water content and subsequent thermal degradation events of the film components. Differential thermogravimetric (DTG) curves were derived to identify the temperatures corresponding to maximum degradation rates, enabling a more detailed comparison of the thermal decomposition profiles. Each measurement was performed in triplicate to ensure data reproducibility and consistency. 

#### 4.7.2. Differential Scanning Calorimetry (DSC) 

Differential scanning calorimetry (DSC, New Castle, DE, USA) was employed to investigate the thermal transitions and potential interactions between the film matrix (polyvinyl alcohol, PVA) and the incorporated niosomal components. The analysis was carried out using a TA Instruments DSC Q1000 calibrated with indium. Samples weighing 5–10 mg were sealed in aluminum hermetic pans and heated from 25 °C to 160 °C at a rate of 10 °C/min under a dry nitrogen purge (flow rate: 50 mL/min). Thermograms were analyzed for the presence of endothermic or glass transition events. The glass transition temperature (Tg) of the PVA matrix was determined using the midpoint method. Particular attention was given to shifts in the Tg value, which were used to assess molecular-level interactions between PVA and the niosomal system. The absence or broadening of melting peaks was interpreted in the context of drug crystallinity or polymer additive interactions. 

### 4.8. Nisin Release Study

The in vitro release of nisin from niosomal formulations and oral film was assessed using the dialysis bag diffusion technique. Pre-activated Thermo Fisher Slide-A-Lyzer™ (Waltham, MA, USA) dialysis cassettes with a molecular weight cut-off (MWCO) of 20,000 Da were used for the study. Each dialysis cassette was loaded with 3 mL of nisin-loaded niosomal formulation, containing 6 mg of nisin, and immersed in 40 mL of distilled water (pH 6.5) as the release medium. The setup was maintained at 37 ± 0.5 °C with constant agitation at 100 rpm in an orbital shaker to simulate physiological conditions. At predetermined time intervals (0, 0.5, 1, 2, 4, 6, 10, 20, 30 and 40), 2 mL aliquots were withdrawn from the release medium and immediately replaced with an equal volume of fresh distilled water to maintain sink conditions. Each collected sample was subsequently mixed with the bicinchoninic acid (BCA) reagent for protein quantification. Samples were incubated for the same duration according to the BCA protocol, and absorbance was measured at 562 nm using a UV–visible spectrophotometer Drawell DU-8600R (Drawell Scientific Instrument, Chongqing, China). Drug concentrations were determined using a previously established calibration curve and the cumulative amount of drug released was calculated accordingly. Corrections were applied at each time point to account for the dilution effect due to media replacement.

### 4.9. Kinetic Model Fitting

Drug release data were fitted to kinetic models using Microsoft Excel by minimizing the residual sum of squares (RSS) between the model predictions and the experimental release profiles. Following this, the coefficient of determination (R^2^) was calculated to assess the correlation between predicted and observed values. Subsequently, the Akaike information criterion (AIC) was determined for each model based on the RSS, using the following equation:

AIC = *n* ln (RSS/*n*) + 2k (2)

where *n* is the number of data points included, k is the number of model parameters, and RSS is the residual sum of squares.

## 5. Results and Discussion

### 5.1. Microfluidic Technology

Table 2 presents the formulation composition and physicochemical properties of various nisin-loaded niosomal systems (NM1–NM5) prepared using microfluidic mixing at two aqueous-to-organic phase ratios (3:1 and 3:2). Each formulation consists of cholesterol (Ch) combined with different surfactants and co-surfactants Span 40, Span 60, Cremophor^®^ RH40 (RH40), and Cremophor^®^ ELP (ELP). The formulations were characterized based on mean vesicle size (nm), polydispersity index (PDI), and encapsulation efficiency (%EE). The data highlight the influence of surfactant type and phase ratio on vesicle uniformity, size distribution, and drug loading capacity, which are critical parameters for optimizing niosomal delivery systems. NM5 (Ch-SP40-RH40) at a 3:1 phase ratio exhibited the smallest particle size (109.1 ± 1.19 nm) with a moderate PDI (0.231 ± 0.013) and an EE% of 25.88%. In contrast, the same formulation at a 3:2 TFR showed a significant increase in size (354.2 ± 3.07 nm) but improved uniformity (PDI = 0.149 ± 0.035) and a slightly higher EE% at 36.57%. Among all formulations, NM2 (Ch-SP60-RH40) at the 3:2 TFR demonstrated the highest EE% (58.33%) with a relatively small particle size (165.3 ± 0.17 nm) and the lowest PDI (0.044 ± 0.020), indicating excellent homogeneity and encapsulation efficiency. This suggests a synergistic effect between SP60 and RH40 in promoting drug entrapment and stable vesicle formation. NM3 and NM4 formulations with ELP as a co-surfactant generally resulted in larger particle sizes and higher PDI values, suggesting less uniform vesicle populations. However, NM4 (Ch-SP60-ELP) at the 3:2 TFR still achieved a relatively high EE of 47.43%, indicating ELP’s potential in enhancing drug loading when used with SP60. In summary, the differences observed across the niosomal formulations can be attributed to variations in surfactant composition, co-surfactants, and the aqueous-to-organic phase ratios. Notably, the combination of cholesterol, Span 60, and RH40 (NM2) at a 3:2 TFR exhibited the most favorable characteristics, with a low particle size (165.3 nm), exceptional homogeneity (PDI = 0.044), and the highest encapsulation efficiency (58.33%). These results can be explained by the high hydrophobicity and long alkyl chain of Span 60, which promotes the formation of stable bilayer structures, while RH40 (a PEG-based surfactant) enhances the vesicle’s surface hydration, reducing aggregation and facilitating better drug entrapment. The NM2 formulation in this study, composed of Span 60, cholesterol, and RH40 at a 3:2 aqueous-to-organic phase ratio, achieved the smallest vesicle size (165.3 ± 0.17 nm), lowest polydispersity index (0.044 ± 0.020), and highest encapsulation efficiency (58.32%) among all tested formulations. Notably, this composition mirrors that of the M2 formulation reported in a vancomycin-loaded niosomal study by Amer et al. which also utilized microfluidic mixing. Their M2 formulation, prepared under identical conditions, demonstrated a particle size of 137.3 ± 0.72 nm, PDI of 0.032 ± 0.024, and EE of 43.12% [18]. These findings suggest that NM2 with a Span 60/cholesterol/RH40 (5: 4: 1) combination at a 3:2 TFR consistently yields nanoscale vesicles with high encapsulation efficiency and favorable size distribution, regardless of the encapsulated peptide. This highlights the robustness and reproducibility of this formulation strategy across different antimicrobial agents.

All experiments were conducted in triplicate (*n* = 3) for each formulation, and results are presented as mean ± SD. Prior to applying parametric tests, data distribution was assessed with the Shapiro–Wilk test for normality and Levene’s test for homogeneity of variance. Both assumptions were satisfied (*p* > 0.05 for all datasets), supporting the use of one-way ANOVA. The one-way ANOVA results provide insight into the effects of both surfactant composition and aqueous-to-organic phase ratio (TFR) on the physicochemical properties of microfluidically prepared niosomal formulations (NM1–NM5). When comparing the five different formulations, no statistically significant differences were observed in particle size (*p* = 0.746, η^2^ = 0.076) or encapsulation efficiency (EE%) (*p* = 0.736, η^2^ = 0.071), indicating that the type of surfactant system used had limited impact on these parameters. However, a statistically significant difference was found in the polydispersity index (PDI) among formulations (*p* = 0.021, η^2^ = 0.413), suggesting that the choice of surfactant and co-surfactant combinations influenced the uniformity of particle size distribution. Additionally, analysis of the impact of TFR (3:1 vs 3:2) revealed no significant effect on particle size (*p* = 0.714, η^2^ = 0.005) or PDI (*p* = 0.176, η^2^ = 0.075), but a statistically significant improvement in EE% was observed with the 3:2 ratio (*p* = 0.0136, η^2^ = 0.231). This enhanced encapsulation is likely due to improved niosome formation efficiency as a result of the higher organic phase content in the microfluidic system, facilitating better drug entrapment.

### 5.2. Antimicrobial Activity

**Figure 4:**
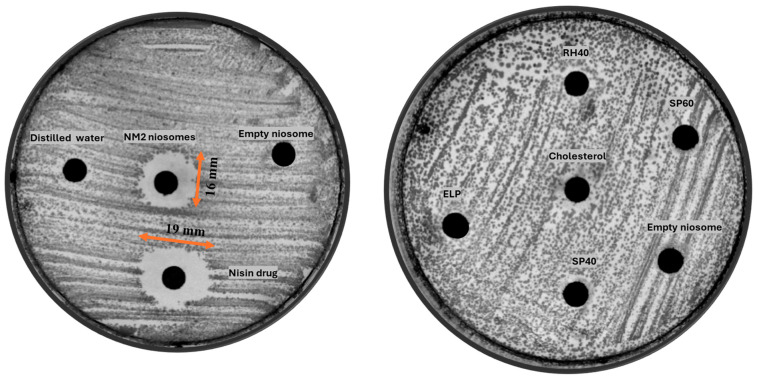
Comparative antimicrobial activity of the optimized NM2 formulation and its components, left plate shows inhibition zones for NM2 (16 mm) and free nisin (19 mm); right plate confirms absence of antimicrobial activity from individual excipients (Span 60, RH40, Span 40, ELP, cholesterol) and blank vesicles. Agar test done in duplicate.

The antimicrobial activity of the optimized NM2 (TFR 3:2) nisin-loaded niosomal formulation was evaluated using an agar diffusion assay and compared against 2 mg/mL free nisin cand control groups (blank niosome of same composition). The zone of inhibition assay is commonly employed as a qualitative or semi-quantitative screening method to assess the antimicrobial activity of formulations. In the context of encapsulated systems, this assay allows for the evaluation of whether the active agent retains its biological activity after encapsulation. The results revealed that free nisin produced a slightly larger inhibition zone (19 mm) than the NM2 (TFR 3:2) formulation (16 mm). This observation is attributable to two key factors (Figure 4). First, the encapsulation efficiency of the NM2 (TFR 3:2) formulation was 58.33%, meaning that only 1.16 mg/mL of the total nisin in the sample was available for diffusion through the agar medium. Second, the encapsulated nisin is released in a controlled manner from the niosomal vesicles, which inherently reduces the rate and extent of diffusion compared to the nisin alone. Therefore, the slightly smaller inhibition zone observed with the NM2 formulation does not necessarily indicate inferior efficacy but rather reflects its modified release behavior and lower immediately available drug content. Furthermore, while nisin alone diffuses rapidly and uniformly, it is also known to be susceptible to enzymatic degradation, particularly in complex biological environments such as the oral cavity [20]. Comparatively, vancomycin-loaded niosomes using the same composition and process (formulation M2, TFR 3:2) demonstrated inhibition zones of 19–25 mm against *Bacillus subtilis* Gram-positive strains [18]. This difference may be attributed to the intrinsic potency of vancomycin, which generally exhibits lower minimum inhibitory concentrations (MICs) than nisin. For example, vancomycin MICs against *S. aureus* typically range from 0.5 to 2 µg/mL, while nisin MICs are often higher, between 8 and 32 µg/mL, depending on the strain and medium conditions [21,22]. In contrast, the niosomal system protects the peptide from such degradation, thereby potentially enhancing its stability and therapeutic persistence at the site of infection. Importantly, no zone of inhibition was observed around the wells containing empty niosomes, indicating that the vesicle components and excipients themselves have no inherent antimicrobial activity. This confirms that the observed antibacterial effect is solely due to the presence of nisin and further supports the biocompatibility and inertness of the niosomal carrier system. Collectively, these findings suggest that while free nisin exhibits greater immediate in vitro diffusion, the NM2 (TFR 3:2) formulation offers a more clinically advantageous delivery profile through sustained release, improved peptide protection, and targeted local action.

The right agar plate further supports this interpretation by demonstrating no measurable antimicrobial activity from individual formulation components (e.g., SP60, RH40, SP40, ELP, cholesterol) or from empty vesicles. This confirms that the surfactants and cholesterol, while crucial for forming stable vesicular structures, do not possess inherent antimicrobial activity under the tested conditions.

### 5.3. Film Characterization

The preliminary evaluation of various film-forming polymers, as presented in Table 3, highlighted significant differences in their film-forming capabilities, mechanical properties, and handling characteristics. Among the four polymers tested, polyvinyl alcohol (PVA) exhibited the most promising performance. Films prepared with PVA were transparent and slightly tacky and displayed high flexibility with good peelability. These favorable characteristics are attributed to PVA’s semi-crystalline nature and its ability to form strong hydrogen bonds, resulting in flexible and mechanically robust films suitable for rapid disintegration formulations [23]. In contrast, hydroxypropyl methylcellulose (HPMC), despite forming clear and smooth films, was visually found to have poor film-forming ability, low flexibility, and high brittleness. The brittleness observed may be due to the high glass transition temperature (Tg) of HPMC, leading to rigid film matrices upon drying, making it unsuitable for flexible oral films. Additionally, HPMC films were difficult to peel from the casting surface, further limiting their practical applicability. Polyvinylpyrrolidone (PVP) films showed moderate film-forming ability and were relatively easy to peel; however, they exhibited significant brittleness and shrinkage upon drying. The shrinkage phenomenon can be explained by the hygroscopic nature of PVP, which tends to absorb water during preparation and lose it rapidly during drying, causing tension and cracks in the film matrix [24]. This compromised the mechanical integrity required for patient handling and administration. Similarly, hydroxypropyl cellulose (HPC) films were found to be sticky after drying, with poor peelability and low flexibility. The stickiness of HPC-based films could be due to its high moisture retention capacity, which prevents proper film setting and drying under normal conditions [25]. Based on these results, PVA was selected as the optimal polymer for further formulation development involving the incorporation of disintegrants and drug-loaded niosomal suspensions. Its excellent film-forming ability, mechanical strength, surface uniformity, and handling characteristics made it the most appropriate candidate for the fabrication of fast-dissolving oral films aimed at enhancing patient compliance and ensuring dose accuracy.

The effect of different disintegrants used less than 1% on the physicomechanical properties and disintegration performance of fast-dissolving films is summarized in Table 4. Among the formulations evaluated, F2 (containing 1% sodium starch glycolate, SSG) exhibited the fastest disintegration time (27 ± 6 s), significantly outperforming both the control film (F4, 113 ± 12 s) and films formulated with MCC (F1, 59 ± 4 s) or COS (F3, 104 ± 2 s). The superior performance of SSG can be attributed to its rapid swelling and wicking properties, which promote faster matrix disruption upon contact with moisture. In fast-dissolving film (FDF) formulations, SSG is commonly used at around 1% *w*/*w*, which provides effective rapid swelling and disintegration [26]. In terms of thickness, all films exhibited comparable values (~0.211 mm), indicating that the incorporation of disintegrants did not significantly alter film thickness. Weight variation among formulations was within 10% variations of uniform dosage forms; however, films containing MCC (F1) showed slightly higher variability (188 ± 30 mg), possibly due to the particulate nature of MCC leading to less uniform film casting and storge high moisture content in drying process. All films maintained a surface pH within the range of 6.5–7.0, which is considered optimal for oral mucosal compatibility, thereby minimizing any risk of mucosal irritation upon application. Folding endurance testing revealed that F2 also had superior mechanical flexibility (77 ± 10 folds before breaking) compared to MCC (44 ± 6) and COS (20 ± 8). Interestingly, the control film (F4) without any disintegrant demonstrated the highest folding endurance (150 folds), suggesting that the absence of disintegrant may enhance mechanical strength but at the cost of much slower disintegration. Overall, the findings indicate free achieving a favorable balance between rapid disintegration and acceptable mechanical strength, making it the optimal choice for further development of fast-dissolving oral films.

All formulations were tested in triplicate (*n* = 3), and results are reported as mean ± SD. Prior to analysis, normality of disintegration times for each group (MCC, SSG, COS, and control) was assessed using the Shapiro–Wilk test (W = 0.954–0.981, *p* = 0.689–0.824), confirming approximate normal distribution. Homogeneity of variance was verified with Levene’s test (*p* = 0.78).

A one-way ANOVA was conducted to evaluate the effect of different disintegrants microcrystalline cellulose (MCC), sodium starch glycolate (SSG), croscarmellose sodium (COS), and a control with no disintegrant on the disintegration time of the formulations, tested in triplicate. The analysis showed a significant difference between groups (*p* < 0.0001) and η^2^ = 0.912, indicating that disintegrant type significantly influences disintegration behavior. Subsequent Tukey’s post-hoc test revealed that the formulation containing SSG exhibited a significantly shorter disintegration time compared to MCC, COS, and the control (*p* < 0.01), while MCC and COS also differed significantly from the control. These results demonstrate the superior performance of SSG in promoting rapid disintegration of fast-dissolving films.

### 5.4. Microscopic Appearance

**Figure 5:**
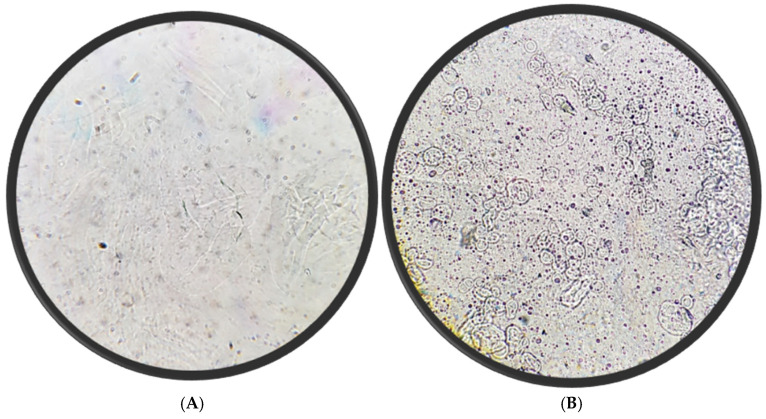
Microscopic evaluation blank oral films (**A**) and nisin niosome film (**B**) dispersed in water (40× magnification).

Figure 5 compares the physical appearance of dissolved oral films without (A) and with (B) nisin-loaded niosomes. The niosome-blank film (A) that contains PVA and PEG400 only appears smooth, transparent, and homogeneous, indicating uniform polymer distribution and the absence of particulate matter. In contrast, the nisin niosome film (B) that contains PVA, PEG, and niosomes displays a well-dispersed population of spherical vesicles, indicating successful rehydration of niosomes from the oral film matrix after dissolution in distilled water. The vesicles retain a predominantly uniform, circular morphology. This difference in appearance suggests successful incorporation of the niosomal suspension and potentially influences the mechanical properties and disintegration profile of the film. The inclusion of vesicles may also enhance the film’s therapeutic functionality by enabling controlled release and protecting nisin from degradation during oral administration.

### 5.5. Scanning Electron Microscope

**Figure 6:**
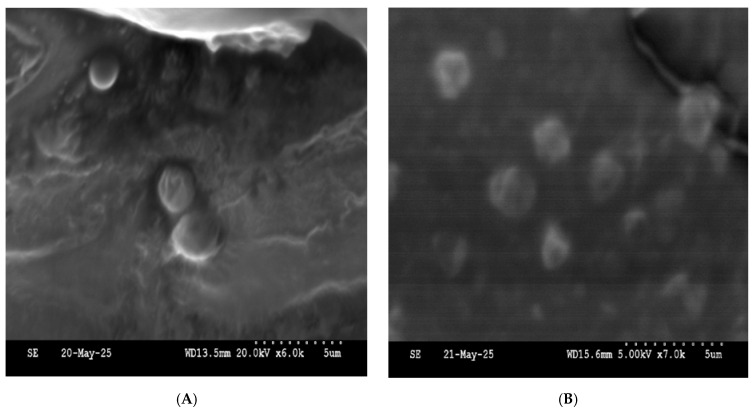
SEM image of NM2 niosomal formulation showing particles. (**A**) magnification ×6000; (**B**) magnification ×7000.

The SEM micrograph of the NM2 niosomal formulation (Figure 6) reveals a heterogeneous population of spherical to slightly oval vesicles with smooth and well-defined surfaces. The vesicles are moderately dispersed, and no significant aggregation or fusion is observed, indicating good formulation stability.

### 5.6. Fourier Transform Infrared Spectroscopy (FTIR) Analysis 

**Figure 7:**
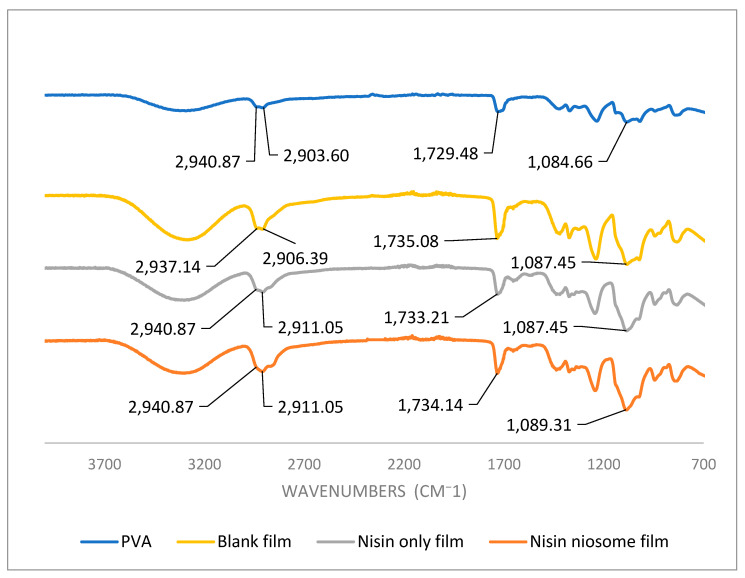
FTIR of the nisin niosome film. Nisin-only film compared to blank film and FTIR of PVA powder.

FTIR was employed to assess potential interactions between the niosome system and the film matrix components. This analysis aimed to detect any chemical or physical interactions, such as hydrogen bonding or compatibility between the excipients and the encapsulated drug. Due to the low concentration of nisin in the final formulation, its characteristic peaks were not prominently detectable or distinguishable in the FTIR spectrum (Figure 7). This is likely attributed to its dilution below the detection threshold of FTIR or overlapping with the dominant spectral signals of other components. The absorption band observed at 2903.6 cm^−1^, corresponding to the C–H stretching vibrations, shows a noticeable shift to 2906.4 cm^−1^ when comparing the pure PVA powder to the blank film, indicating an interaction induced by the film formation process [27,28]. The same shift is observed in the nisin-loaded film, suggesting continued structural changes when nisin is introduced to the system increasing the p–p interactions. Additionally, the peak at 1729.5 cm^−1^, associated with C=O stretching vibrations, also shifts from the pure PVA powder to the blank film, supporting the presence of interactions likely involving polyethylene glycol (PEG) [27,28]. In contrast, only a minor shift is observed between the blank film and the films containing nisin or nisin-loaded niosomes, indicating that the addition of nisin does not significantly alter the carbonyl environment within the film matrix. Furthermore, the absorption band at 1087.45 cm^−1^, attributed to C–O stretching, displays a clear shift in the blank film compared to the PVA powder. However, no substantial shifts are detected when comparing the blank film to the nisin and nisin-loaded formulations, reinforcing the conclusion that the chemical environment of the C–O bond remains largely unchanged with the addition of nisin. Collectively, these spectral changes suggest that the observed peak shifts are primarily due to interactions between PVA and PEG, particularly during film formation. The minimal additional shifts upon the incorporation of nisin or nisin-loaded niosomes indicate that no significant molecular interactions occur between the nisin formulations and the PVA/PEG film matrix, implying that nisin is physically embedded rather than chemically interacting with the polymer network.

### 5.7. Thermal Analysis Results

#### 5.7.1. Thermogravimetric Analysis

**Figure 8:**
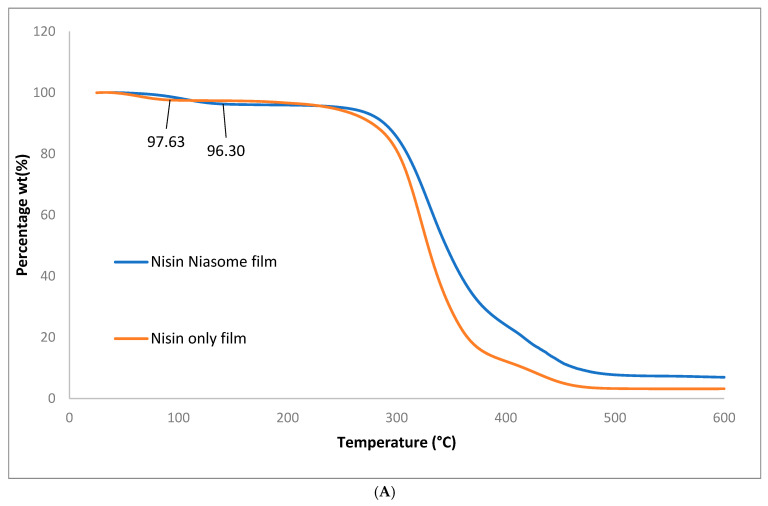

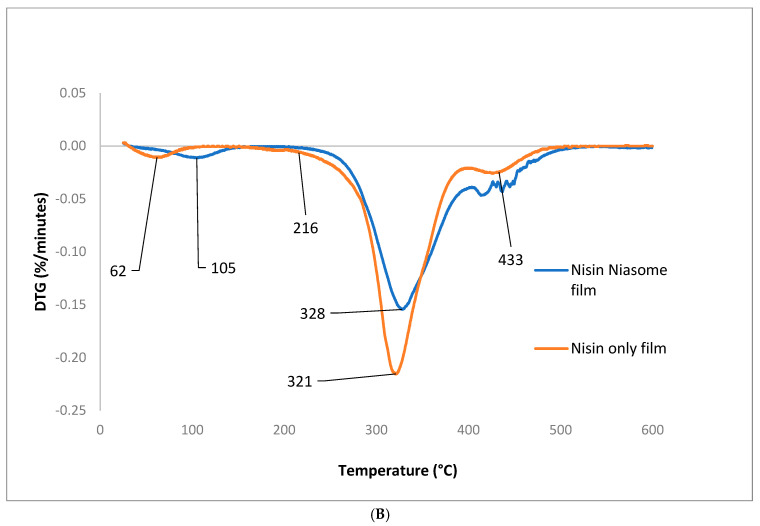
Thermal gravimetric analysis (**A**) and differential thermogravimetry (**B**) for nisin niosome and nisin-only film.

Thermogravimetric analysis (TGA) was conducted to investigate the thermal stability and moisture content of the niosome-based nisin film formulation (Figure 8). The results revealed that the niosome-containing film exhibited a slightly higher initial mass loss attributed to water content 3.7% compared to 2.37% in the nisin-only film. This elevated moisture content is likely due to the aqueous core of the niosomal vesicles, which retain water within their bilayer structure. Further insight was provided by the differential thermogravimetric (DTG) curve, where the first thermal event, associated with the evaporation of water, showed an increase in peak intensity and a slight shift to a higher temperature in the niosome formulation. This behavior suggests that water is more tightly bound or encapsulated in the vesicular structure of the niosomes, requiring greater thermal energy to be released, which aligns with the hypothesis of water entrapment within the formulation matrix. PVA is composed of repeating units of vinyl alcohol. Its chemical structure consists of a carbon–carbon backbone with hydroxyl (–OH) groups attached to alternating carbon atoms: [−CH2–CH(OH)−]_n_. Therefore, the initial degradation step of polyvinyl alcohol (PVA), typically associated with the elimination of water from the polymer backbone, occurred at a higher temperature in the niosome-based film (328 °C) compared to the nisin-only film (321 °C). This thermal shift, along with the broader degradation peak observed in the DTG curve, suggests that the incorporation of the niosomal system contributes to the thermal stabilization of the polymer matrix. The broader peak may also indicate a more gradual degradation process, possibly due to increased intermolecular interactions such as hydrogen bonding between the PVA and the niosomal components. Collectively, these thermal analyses support the conclusion that the niosome formulation enhances the structural stability of the film by retaining more water and delaying the thermal degradation of PVA, which may have implications for the mechanical properties, shelf-life, and drug release behavior of the final product. 

#### 5.7.2. Differential Scanning Calorimetry

Differential scanning calorimetry (DSC) was used to investigate the thermal behavior of the film formulations to identify any possible interactions between the film matrix, primarily composed of PVA, and the niosomal components. Nisin pure powder did not display a sharp endothermic melting peak, which is consistent with its amorphous nature. PVA powder shows Tg 42.4 °C which is different from what is reported in the literature (75–85 °C) [29] due to the presence water moisture which act as plasticizer lowering the Tg of the polymer. However, the blank film experiences a higher Tg related to the removal of the moisture content during the film preparation. Moreover, a notable shift was observed in the glass transition temperature (Tg) of the PVA matrix. The Tg decreased from 94.7 °C in the nisin-only film to 66.5 °C in the film containing nisin-loaded niosomes. This reduction in Tg indicates a possible plasticizing effect or molecular-level interaction between the PVA polymer chains and the nisin and niosomal components. These results align with FTIR results which show the same interaction between the nisin and the film components. 

### 5.8. Film Tensile Strength 

**Figure 9:**
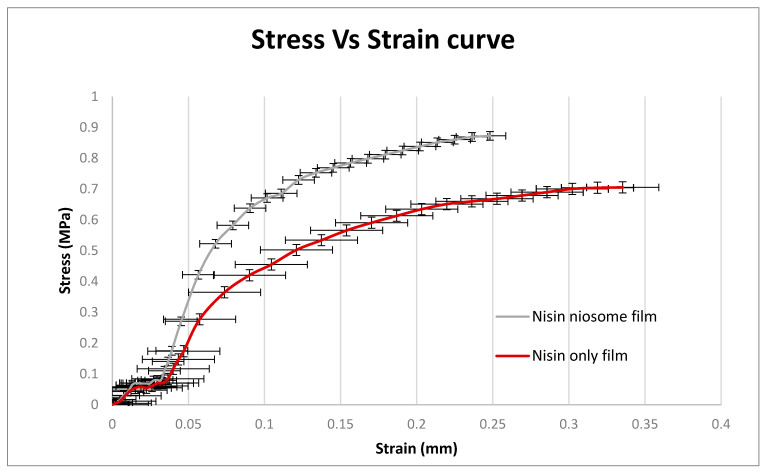
Stress against strain curve for tensile strength of the nisin-loaded niosome film and nisin-only film (*n* = 3).

Tensile strength analysis revealed notable differences in the mechanical properties between the nisin-only film and the nisin-loaded niosome film. The nisin-only film exhibited a higher strain at break (0.3078 mm) compared to the nisin-loaded niosome film (0.2152 mm), indicating that the former had greater elasticity and was able to stretch more before breaking (Figure 9). However, the maximum stress required to break the nisin-only film was lower (0.7190 MPa) than that of the nisin-loaded niosome film (0.8879 MPa), suggesting that the incorporation of niosomes enhanced the film’s mechanical strength, which can be the result of the interaction between the niosome component and PVA in the film formulation. Statistical analysis confirmed that these differences in both stress and strain were significant, with a p-value of 0.0002 for stress and 0.0035 for strain. These findings suggest a trade-off between elasticity and tensile strength depending on the film composition, where the nisin-only film favors flexibility while the niosome formulation contributes to improved resistance to mechanical failure. Additionally, a significant difference in Young’s modulus between the nisin-only film and the nisin-loaded niosome film. The nisin-only film exhibited a Young’s modulus of 2.437 Pa, indicating a relatively flexible and less rigid structure. In contrast, the nisin-loaded niosome film demonstrated a markedly higher modulus of 4.238 Pa, suggesting improved stiffness and mechanical strength. 

### 5.9. In Vitro Release Profile

**Figure 10:**
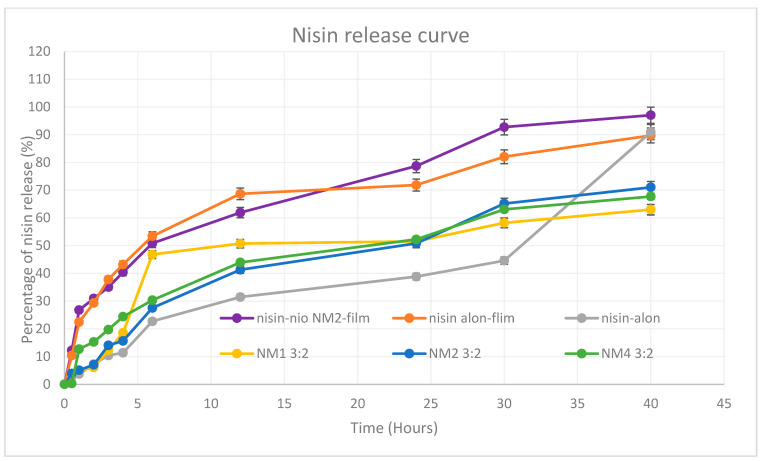
In vitro cumulative percentage release profiles of nisin from film-based and microfluidic niosomal formulations (mean ± SD, *n* = 3). This figure presents the in vitro cumulative release of nisin from two film-based delivery systems nisin-loaded niosome NM2-film (niosomal encapsulated nisin) and nisin-only film (free nisin incorporated into film) and three microfluidic-prepared niosomal suspensions NM1, NM2, and NM4 with a total flow rate ratio of 3:2.

The in vitro release profile of nisin was evaluated for five formulations (Figure 10) over a 40-h period using a dialysis cassette (Thermo Fisher, 20,000 MWCO) and the results reveal clear differences in release profiles depending on the formulation approach and composition. These included two film-based formulations, namely nisin-loaded niosome NM2 film and nisin-only film, and three niosome-based systems prepared using microfluidics with 3:2 total flow rate, including NM1 (Ch-SP60-RH40, 35:45:20), NM2 (Ch-SP60-RH40, 50:40:10), and NM4 (Ch-SP60-ELP, 35:45:20). All niosome formulations contained both encapsulated and free drug. The film-based formulations demonstrated a sustained and efficient release. The nisin-loaded niosome NM2-film, incorporated with NM2 niosomes formula within a polymer matrix of PVA (31 kDa) and PEG 400, exhibited the most rapid and the highest overall release (97.03% at 40 h), indicating a synergistic effect between the film and niosomal encapsulation. PEG 400, a known solubilizing agent and plasticizer, likely improved the aqueous solubility of nisin by increasing matrix hydrophilicity and promoting faster diffusion from the film [30]. This enhanced solubility may also have facilitated quicker release of both entrapped and non-entrapped drug components, especially in the initial hours. The nisin-only film followed closely with 89.73% release, likely reflecting the release of unencapsulated drug dispersed within the polymer. These film formulations provided an initial burst within the first 4–6 h (~50%) followed by a sustained release phase, highlighting their potential for buccal or mucosal delivery applications where prolonged exposure is desirable. These findings indicate that burst release is not only dependent on nisin entrapment efficiency but is also influenced by the molecular-level interactions between film polymers and surfactants. Therefore, the initial pursed release is attributed to the weaker or transiently associated free nisin.

Among the niosomal suspensions, NM2 showed the most controlled and the highest cumulative release (71.02% at 40 h). This is likely attributed to its smallest vesicle size (165.3 ± 0.17 nm), narrowest PDI (0.044), and highest entrapment efficiency (EE%, 58.33%). Smaller, uniform vesicles can ensure better diffusion across the dialysis membrane and controlled drug liberation. NM4 exhibited moderate release (67.72%), possibly influenced by its larger particle size (230.1 ± 14.29 nm) and broader PDI (0.358), which may indicate structural heterogeneity and slower diffusion. NM1 showed the lowest overall % release among the niosomes (62.99%), which may be attributed to its lower EE% (36.9%) and intermediate size (203.3 ± 1.29 nm) and the faster release for the first 5 h is due to the amount of unencapsulated nisin in the formula. The unformulated nisin control (nisin only) in solution exhibited an initial lag in release (only 2.74% at 0.5 h and 11.35% at 4 h), likely due to binding interactions, diffusion resistance or missing of any solubilizing facilitator from PEG 400 in oral film formulation or non-ionic surfactant in niosomes through the dialysis membrane. However, it showed a sharp increase after 30 h, reaching ~91% at 40 h, consistent with unencapsulated drug accumulation over time. The release study presented here focuses on nisin, which is not a direct reflection of what will occur in the oral cavity. For the oral film, we expect complete disintegration in under two minutes (as confirmed by the disintegration test). Although in vitro release of nisin required up to 40 h to reach completion, we anticipate that in the oral environment of the niosomal nanoparticles will be released much earlier, providing the therapeutically active form within the short residence time of the film. Incorporating niosomes will also facilitate and improve penetration of nisin through bacterial cell walls, thereby enhancing efficacy. In contrast, when a film containing only free nisin is used, the peptide is more likely to be rapidly washed away by saliva before effective interaction with bacterial cells can occur. Furthermore, the nanoscale encapsulation is expected to enhance the stability of nisin against enzymatic degradation, thereby prolonging its antimicrobial effectiveness in the oral cavity. In summary, encapsulating nisin within NM2-based niosomes, especially when incorporated into film matrices, significantly enhanced the release profile in terms of both rate and extent. The superior performance of the nisin-loaded niosome NM2-film is attributed to the combined benefits of high EE%, small vesicle size, and polymer-facilitated sustained release. These findings highlight the potential of tailored niosome-polymer hybrid systems for mucosal or controlled drug delivery of peptide therapeutics like nisin.

### 5.10. Kinetic Modelling of Drug Release

The release profiles of nisin alone, nisin encapsulated in niosomes, and nisin–niosomes incorporated into films were evaluated using several kinetic models as shown in (Table 5) to identify the most appropriate release mechanism. The zero-order model was examined to assess concentration-independent release, characteristic of sustained-release systems. The first-order model was applied to represent concentration-dependent release, often observed in immediate-release formulations. The Higuchi model was used to describe diffusion-controlled release in polymeric matrices, while the Hopfenberg model was considered for release governed by surface erosion of the polymer matrix. Additionally, the Korsmeyer–Peppas model was employed as a semi-empirical approach to characterize the release mechanism through the release exponent (*n*), which distinguishes among Fickian diffusion, anomalous (non-Fickian) transport, and Case II transport [31].

To determine the best-fitting model for each system, two evaluation criteria were applied: the correlation coefficient (R^2^) and the Akaike information criterion (AIC). While R^2^ indicates the degree of correlation between observed and predicted data, it may favor overfitting. Conversely, AIC accounts for model complexity by penalizing additional parameters, making it more suitable for comparing models across different datasets. Both metrics were therefore employed in parallel to ensure a robust selection of the most appropriate release model [31].

For nisin alone, the release profile was best described by the zero-order (R^2^ = 0.955, AIC = 44.55) and Korsmeyer–Peppas models (R^2^ = 0.952, AIC = 45.50), suggesting a nearly concentration-independent diffusion process. In contrast, the nisin-loaded film followed first-order kinetics (R^2^ = 0.983, AIC = 36.63), indicating a concentration-dependent release, consistent with diffusion across the film matrix. The nisin-loaded niosomes prior to film incorporation (NM1, NM2, and NM4) displayed a different release behavior. NM1 was best fitted to the first-order model (R^2^ = 0.964, AIC = 41.10) with a Korsmeyer–Peppas release exponent (*n* = 0.48), pointing to anomalous transport governed by both diffusion and matrix relaxation. Similarly, NM2 showed an excellent fit to the first-order model (R^2^ = 0.992, AIC = 26.04), with comparable fits to the Higuchi and Korsmeyer–Peppas models (R^2^ = 0.991 and 0.990, respectively), and an exponent *n* = 0.61, further supporting anomalous transport. In contrast, NM4 showed a closer alignment with the Higuchi model (R^2^ = 0.987, AIC = 26.75) and a release exponent of *n* = 0.48, indicating a predominantly diffusion-controlled mechanism. This shift in release behavior can be attributed to the use of a different co-surfactant, Kolliphor^®^ ELP, in the preparation of NM4, compared to Kolliphor^®^ RH40 employed in NM1 and NM2. Interestingly, once NM2 was incorporated into the film, the release profile shifted markedly to the Korsmeyer–Peppas model (R^2^ = 0.994, AIC = 25.39), with an exponent *n* = 0.39, confirming a Fickian diffusion-controlled mechanism. Collectively, these findings suggest that while free nisin undergoes rapid diffusion and niosomal formulations release through anomalous transport involving both diffusion and erosion, incorporation of niosomes into films imposes an additional diffusion barrier, thereby converting the release mechanism into controlled Fickian diffusion. 

## 6. Conclusions and Future Work

In this study, fast-dissolving oral films incorporating nisin-loaded niosomes were successfully formulated and optimized for potential antimicrobial therapy. Niosomes were prepared using a combination of microfluidic mixing and thin-film hydration techniques, allowing for the production of nanoscale vesicles with high uniformity, controlled size, and efficient encapsulation of nisin. Physicochemical evaluation demonstrated that the optimized niosomal formulation exhibited favorable particle size, low polydispersity index (PDI), and high encapsulation efficiency, supporting its suitability for incorporation into oral film systems.

Polymer screening identified polyvinyl alcohol (PVA) as the most suitable film-forming polymer, producing films with excellent flexibility, good transparency, and minimal brittleness. Further optimization using different disintegrants revealed that films formulated with sodium starch glycolate (SSG) exhibited the most rapid disintegration (27 ± 6 s) while maintaining acceptable folding endurance and mechanical strength. Importantly, all film formulations maintained a surface pH between 6.5 and 7.0, which is compatible with oral mucosal tissues, thus reducing the risk of irritation.

Furthermore, antimicrobial testing using agar diffusion assays demonstrated that both free nisin and nisin-loaded niosomal films exhibited significant antibacterial activity, with observable inhibition zones against targeted bacterial strains. Although the free nisin exhibited a slightly larger inhibition zone compared to the niosomal formulation while the concentration of nisin alone is 2 mg/mL, the controlled release behavior and enhanced stability offered by the niosomal encapsulation suggest improved therapeutic potential for sustained action in vivo.

In summary, the comprehensive physicochemical and mechanical characterization of the developed films confirms their potential suitability for oral application. The uniformity in weight and thickness ensured batch-to-batch consistency, while hand-folding and tensile testing highlighted the influence of niosomal incorporation on mechanical performance most notably in enhanced tensile strength and Young’s modulus, indicating improved film rigidity and structural integrity. FTIR and DSC analyses revealed subtle yet meaningful interactions between the PVA matrix and the niosomal components, particularly evidenced by shifts in functional group peaks and glass transition temperatures. These changes suggest physical embedding and possible molecular interactions without chemical modification of nisin. Moreover, thermal analysis via TGA/DTG showed increased thermal stability and moisture retention in the niosome-loaded films, supporting their enhanced structural robustness. Collectively, these findings underscore the role of the niosomal system in modulating film properties and demonstrate the potential of this formulation strategy for stable, bio-functional oral film delivery platforms.

The film matrix serves as a structural scaffold that encapsulates and retains the nanoparticles, thereby contributing to enhanced stability by reducing aggregation and protecting the active agent from environmental stress. To fully assess the robustness of the formulations, comprehensive stability studies under varying temperature and humidity conditions will be conducted in future investigations.

Overall, the developed nisin-loaded niosomal fast-dissolving oral films offer a promising platform for localized antimicrobial therapy within the oral cavity. These films combine the advantages of rapid disintegration, mucosal compatibility, and enhanced peptide stability, positioning them as attractive candidates for treating oral infections, dental biofilms, and potentially systemic infections originating from oral bacterial colonization. Further in vivo studies are warranted to validate their clinical efficacy and therapeutic applicability.

## Figures and Tables

**Table 1:** Non-ionic surfactant composition of niosomal formulations (percentage as molar ratios).

Formulation	Niosomes Composition	Cholesterol	Span 60	Span 40	Kolliphor RH40	Kolliphor ELP
NM1	Ch-SP60-RH40	35%	45%		20%	
NM2	Ch-SP60-RH40	50%	40%		10%	
NM3	Ch-SP40-ELP	35%		45%		20%
NM4	Ch-SP60-ELP	35%	45%			20%
NM5	Ch-SP40-RH40	35%		45%	20%	

**Table 2:** Characterization of niosomal formulations of nisin prepared using microfluidic mixing: size, PDI, and encapsulation efficiency.

Formulation	Niosome Composition	Aqueous to Organic Phase Ratio	Size (nm)	PDI	EE%
NM1	Ch-SP60-RH40	3:1	140.9 ± 1.6	0.227 ± 0.005	23.41
		3:2	203.3 ± 1.3	0.198 ± 0.024	36.90
NM2	Ch-SP60-RH40	3:1	145.1 ± 1.2	0.145 ± 0.009	25.74
		3:2	165.3 ± 0.2	0.044 ± 0.020	58.32
NM3	Ch-SP40-ELP	3:1	219.0 ± 1.5	0.269 ± 0.014	21.78
		3:2	236.1 ± 3.4	0.316 ± 0.019	40.47
NM4	Ch-SP60-ELP	3:1	260.9 ± 2.7	0.313 ± 0.063	40.21
		3:2	230.1 ± 14.3	0.358 ± 0.010	47.43
NM5	Ch-SP40-RH40	3:1	109.1 ± 1.2	0.231 ± 0.013	25.87
		3:2	354.2 ± 3.0	0.149 ± 0.035	36.57

**Table 3:** Comparative evaluation of film-forming ability, mechanical properties, and visual characteristics of four polymers (HPMC, PVA, PVP, HPC) for oral film formulation. Films prepared in triplicate.

Polymer	Film-Forming Ability	Visual Appearance	Flexibility	Peelability	Brittleness	Selected for Further Optimization	Film Shape and Surface Uniformity
HPMC	Poor	Clear, smooth	Low	Poor	High	No	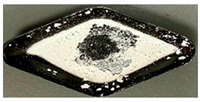
PVA	Excellent	Transparent, slightly tacky	High	Good	Slight	Yes	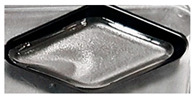
PVP	Moderate	Brittle, shrinks on drying	Low	Good	High	No	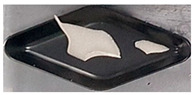
HPC	Poor	Sticky on drying	Low	Poor	None	No	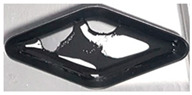

**Table 4:** Comparative evaluation of microcrystalline cellulose, sodium starch glycolate, and croscarmellose sodium on the disintegration and mechanical properties of oral fast-disintegrating films.

Formulation Code	Disintegrant	Disintegration Time (s)	Thickness (mm)	Weight Variation (mg)	Surface pH	Folding	Surface Uniformity
F1	Microcrystalline Cellulose (MCC)	59 ± 4	0.211 ± 4	188 ± 30	6.5–7.0	44 ± 6	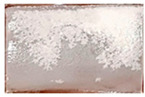
F2	Sodium Starch Glycolate (SSG)	27 ± 6	0.211 ± 4	173 ± 8	6.5–7.0	77 ± 10	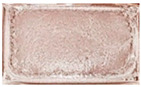
F3	Croscarmellose Sodium (COS)	104 ± 2	0.201 ± 4	165 ± 20	6.5–7.0	20 ± 8	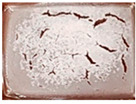
F4	None	113 ± 12	0.211 ± 4	160 ± 2	6.5–7.0	150	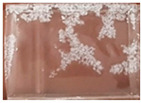

**Table 5:** Kinetic modeling of nisin release from niosomal and film formulations: R^2^, AIC, and release mechanisms.

Kinetic Model		Nisin-Loaded Niosome NM2-Film	Nisin-Only Film	Nisin Only	NM1 3:2	NM2 3:2	NM4 3:2
**Zero order**	**R2**	0.954	0.905	0.955	0.857	0.968	0.946
	**AIC**	47.510	51.462	44.553	53.604	39.995	42.975
**First order**	**R2**	0.971	0.983	0.838	0.964	0.992	0.986
	**AIC**	45.253	36.636	71.497	41.106	26.045	30.475
**Higuchi**	**R2**	0.991	0.966	0.932	0.923	0.991	0.987
	**AIC**	42.523	47.018	64.706	45.890	33.530	26.758
**Hopfenberg**	**R2**	0.967	0.984	0.836	0.966	0.965	0.980
	**AIC**	49.257	47.316	71.179	42.812	45.306	35.582
**Korsmeyer–Peppas model**	**R2**	0.994	0.981	0.952	0.925	0.990	0.988
	**AIC**	25.389	36.128	45.498	47.826	28.966	46.130
**Korsmeyer–Peppas model release exponent (*n*)**		0.390	0.352	0.881	0.480	0.611	0.481

## Data Availability

Data is contained within the article.
